# Efficacy and Safety of Toludesvenlafaxine Hydrochloride Sustained-Release Tablets in Depression With Anhedonia: A Single-Arm, Multicenter Clinical Study

**DOI:** 10.1155/da/6130764

**Published:** 2025-05-05

**Authors:** San-wang Wang, Wei-feng Mi, Xiao-nan Hao, Xiao-xing Liu, Xin Wen, Min Zhao, Hai-feng Jiang, Wen-zheng Wang, Tao Li, Zhong-Lin Tan, Song Chen, Wen Lv, Yu-ping Ning, Yan-ling Zhou, Ying-mei Chen, Xiang-dong Tang, Bin Li, Yang Liu, Xian-cang Ma, Ying–ying Dong, Yun-chun Chen, Hui-ling Wang, Yong-lan Huang, Hua Zhang, Lin Lu

**Affiliations:** ^1^Department of Psychiatry, Renmin Hospital of Wuhan University, Wuhan, China; ^2^Peking University Sixth Hospital, Peking University Institute of Mental Health, NHC Key Laboratory of Mental Health (Peking University), National Clinical Research Center for Mental Disorders (Peking University Sixth Hospital), Beijing, China; ^3^Peking-Tsinghua Center for Life Sciences and PKU-IDG/McGovern Institute for Brain Research, Peking University, Beijing, China; ^4^Shanghai Mental Health Center, Shanghai Jiao Tong University School of Medicine, Shanghai, China; ^5^Affiliated Mental Health Centre and Hangzhou Seventh People's Hospital, Zhejiang University School of Medicine, Hangzhou, Zhejiang, China; ^6^Department of Psychiatry, The Affiliated Brain Hospital of Guangzhou Medical University, Guangzhou, China; ^7^Mental Health Center, West China Hospital, Sichuan University, Chengdu, China; ^8^Department of Psychiatry, The First Affiliated Hospital of Xi'an Jiaotong University, Xi'an, China

**Keywords:** anhedonia, depression, multicenter clinical study, toludesvenlafaxine

## Abstract

Toludesvenlafaxine hydrochloride sustained-release tablets, as China's first independently developed chemical Class 1 innovative drug with independent intellectual property rights for the treatment of depression and a new molecular entity, represent a novel triple reuptake inhibitor (TRI) with specific target selectivity for serotonin (5-HT), norepinephrine (NE), and dopamine (DA). This single-arm, multicenter clinical study aimed to evaluate the efficacy and safety of toludesvenlafaxine in alleviating anhedonia symptoms in patients with major depressive disorder (MDD). A total of 123 patients aged 18–65 years were enrolled between April 2023 and April 2024 and received an 8-week treatment with toludesvenlafaxine sustained-release tablets (80–160 mg/day). The primary efficacy endpoint was the change in the total score of the Dimensional Anhedonia Rating Scale (DARS) at weeks 2, 4, and 8. Significant improvements in DARS scores were observed, with mean changes from baseline of 8.4 (95% CI [6.4, 10.4], *p* < 0.0001), 14.1 (95% CI [12.0, 16.2], *p* < 0.0001), and 20.4 (95% CI [18.0, 22.9], *p* < 0.0001), respectively. Additionally, after 8 weeks of treatment, plasma levels of neurotrophic factors, including mature brain-derived neurotrophic factor (mBDNF) (*t* = 28.78, *p* < 0.0001), pro-BDNF (*t* = 27.71, *p* < 0.0001), and vascular endothelial growth factor (VEGF) (*t* = 31.07, *p* < 0.0001), were significantly increased, and the plasma level of IGF-1 was not significantly changed (*t* = 0.35, *p*=0.7269). No association was found between the percentage of changes in neurotrophic factors and the percentage of symptom improvements. Toludesvenlafaxine was generally well-tolerated, with treatment-emergent adverse events (AEs) (TEAEs) reported in 83.7% of participants and treatment-related AEs (TRAEs) in 76.4%. These findings indicate that toludesvenlafaxine hydrochloride sustained-release tablets are safe, well-tolerated, and effective in alleviating anhedonia symptoms in patients with depression.

**Trial Registration:** http://www.chictr.org.cn (No.: ChiCTR2300070331).

## 1. Introduction

Major depressive disorder (MDD) is the leading cause of disability worldwide, affecting more than 300 million people worldwide [1, 2]. Anhedonia, or loss of interest or pleasure in all or almost all activities, is the hallmark symptom of depressive disorders [3]. It has been shown to be present in 50%–80% of individuals with depression [4]. Notably, anhedonia has been proven to be a predictor of poor prognosis of antidepressant treatment; depressed patients with anhedonia may have more serious symptoms and higher suicide risks [5]. First-line treatments for depressive disorders, including tricyclic antidepressants, monoamine oxidase inhibitors, selective serotonin reuptake inhibitors (SSRIs), serotonin and norepinephrine (NE) reuptake inhibitors (SNRIs), and serotonin (5-HT) receptor partial agonist/reuptake inhibitors, can effectively alleviate depressive symptoms. However, their therapeutic effects on anhedonia are limited due to the lack of mechanism-based modulation, necessitating the definition of novel therapeutic approaches based on the mechanism of anhedonia [6].

The pathology underlying the development of anhedonia is complex. Current research suggests that the development of anhedonia is closely related to the dysfunction of the brain reward system [7, 8]. Parallelly, numerous studies have identified the critical role of the mesolimbic dopaminergic (dopamine [DA]) circuit in the reward system [9]. The DA mesolimbic pathway originates in the ventral tegmental area (VTA) and projects to the ventral (nucleus accumbens [NAc]) and dorsal (caudate and putamen) striatum. It then proceeds to the orbitofrontal cortex (OFC), several anterior cingulate cortex (ACC) subregions, and more dorsal aspects of the prefrontal cortex (PFC). The medial forebrain bundle is a white matter tract that anatomically connects the primary reward system regions (e.g., VTA → NAc), which has been firmly linked to the perception of pleasure and motivated behavior [10, 11]. Preclinical studies have emphasized the DA neural activity in mediating the development of anhedonia. For instance, optogenetic phasic stimulation of VTA DA neurons quickly induced anhedonic phenotype in susceptible mice as measured by decreased sucrose preference, whereas optogenetic inhibition of VTA-NAc DA neurons reversed anhedonia [12]; in the other aspect, optogenetic stimulation of NAc-projecting DA neurons during social defeat promoted resilience [13]. In human research, it was found that genetic polymorphisms in proteins regulating DA synthesis, metabolism, and functional activity correlate with functional activity in brain regions associated with reward processing and are further associated with the clinical features of anhedonia in patients with depression [14, 15].

Collectively, these studies suggest that DA neural pathways may be a potential target for drug development for depression patients with anhedonia. Previous studies have demonstrated that novel antidepressants (agomelatine and vortioxetine) and bupropion can significantly alleviate anhedonia in depressed patients, and the symptom improvements are closely related to the direct or indirect modulation of the DA system [16–18]. In addition, a study completed by the Canadian Biomarkers Integration Network for Depression (CAN-BIND-1) found that 61% of patients with major depression who did not respond to escitalopram showed significant improvements in anhedonia with the adjunctive use of DA drug aripiprazole [19]. These evidence-based data all implicate that the mutual synergistic effect of 5-HT, NE, and DA systems is crucial for alleviating the anhedonia in depression. Moreover, a reduction in the expression of neurotrophic factors such as brain-derived neurotrophic factor (BDNF) and vascular endothelial growth factor (VEGF), along with decreased receptor activity, represents a significant alteration associated with depression. Studies have shown that the levels of VEGF are significantly reduced in patients with MDD [20]. Notably, recent research indicated that depressed patients with anhedonia exhibited imbalances in plasma BDNF, with an increased ratio of mature BDNF (mBDNF) to BDNF precursor (proBDNF) correlating with the severity of anhedonia [21]. Additionally, studies showed that the plasma level of another neurotrophic factor, insulin-like growth factor 1 (IGF-1), was significantly higher in MDD patients compared to healthy controls and may be related to the pathology of depression [22, 23]. Furthermore, DA has been shown to modulate the expression levels of neurotrophic factors [24, 25]. Therefore, it is worth investigating whether DA treatments can improve anhedonia by regulating the expression levels of neurotrophic factors.

Toludesvenlafaxine hydrochloride sustained-release tablets, as China's first independently developed chemical Class 1 innovative drug with independent intellectual property rights for the treatment of depression and a new molecular entity, is a novel triple reuptake inhibitor (TRI) drug with specific target selectivity for 5-HT, NE, and DA. Affinity tests have demonstrated that toludesvenlafaxine has a high affinity for the human 5-hydroxytryptamine transporter (serotonin transporter [SERT]), the NE transporter (NET), and the DA transporter (DAT) and in particular has about 31-fold higher affinity than that of venlafaxine for the DAT in the same experimental conditions [26]. Besides, in vitro cellular assay showed that toludesvenlafaxine had reuptake inhibitory effects on 5-HT, NE, and DA, with IC50s of 31.40 ± 0.43 nM, 586.70 ± 83.57 nM, and 733.20 ± 10.26 nM, respectively, whereas the control drug, desvenlafaxine succinate (DVS), had reuptake inhibitory effects only on 5-HT and NE, with an IC50 of 53.46 ± 7.7 nM, and an IC50 of 53.46 ± 7.7 nM, respectively [27]. Moreover, single or multiple doses of Rohypnol significantly increased the levels of 5-HT, NE, and DA in the synaptic gap of social-defeated rats, while DVS did not affect the increase of DA concentration [28].

Preclinical study demonstrated that toludesvenlafaxine hydrochloride sustained-release tablets significantly increased the sugar–water preference index in depression model rats, reflecting a significant improvement in anhedonia [27]. In addition, the most recent phase III clinical trial found toludesvenlafaxine at both 60 mg/day and 180 mg/day was effective and safe in the treatment of depression and improved symptoms of anhedonia in depressed patients [29]. However, this study did not use a specific scale for the assessment of the anhedonia; thus, the credibility of the results needs to be confirmed. The relationship between the improvement in anhedonia and patients' overall functioning and quality of life also remains to be explored. Therefore, to further assess the efficacy and safety of toludesvenlafaxine hydrochloride sustained-release tablets for the treatment of anhedonia in patients with depression, we conducted this multicenter, single-arm clinical study using a specific scale for anhedonia based on a phase III clinical trial.

## 2. Methods

### 2.1. Study Design

This study was a single-arm, multicenter clinical study, which was conducted in seven hospitals in China from April 2023 to April 2024 after receiving a multicenter independent ethics committee approval. This study was performed in strict compliance with the principles of Good Clinical Practice, the Declaration of Helsinki, and other relevant regulations. All participants were enrolled in the study after providing written informed consent. Researchers at each center received standardized training to ensure consistency in the quality of the study across centers. The study was registered on http://www.chictr.org.cn (No.: ChiCTR2300070331).

### 2.2. Patients

Patients were eligible for enrollment if they were 18–65 years of age and had clinically significant anhedonia as defined by the Chinese version of the Dimensional Anhedonia Rating Scale (DARS) at a screening score of less than or equal to 28.5 [30]. In addition, participants had to meet the DSM-5 diagnostic criteria for MDD on the basis of the Mini-International Neuropsychiatric Interview (MINI 7.0) for DSM-5 and were currently experiencing a moderate to severe depressive episode (scores ≥26 on the Montgomery–Ãsberg Depression Rating Scale [MADRS]).

Participants were excluded if they met the DSM-5 diagnosis of any disorder other than depression; met DSM-5 criteria for substance-related or alcohol use disorder (other than nicotine or caffeine) within 6 months prior to screening; had severe self-injury behavior, apparent suicide attempts or behavior, a score ≥4 on the item 10 of the MADRS (suicide ideation); had depressive episode secondary to other psychiatric or somatic disorders; had a history of seizures (except convulsions caused by pediatric febrile convulsions); used any antidepressants or mood stabilizers in the last 2 weeks; were allergic or had known hypersensitivity to venlafaxine and desvenlafaxine; failed to respond to venlafaxine or at least two different types of antidepressants in the previous treatment; used sleep aids such as benzodiazepines in the last 3 days; used fluoxetine and vortioxetine in the last 1 month; were pregnant or lactating, had recently planned to become pregnant, or used contraceptives; had clinical abnormalities on total bilirubin (TBIL), alanine aminotransferase (ALT) or aspartate aminotransferase (AST), creatinine, thyroid-stimulating hormone (TSH), or 12-lead electrocardiogram (ECG) at screening period; had other serious acute or chronic diseases, psychiatric disorders, or clinically evident abnormal laboratory examination; and were identified as not suitable to participate in this study by the investigators.

### 2.3. Procedures

The study comprised a 1-week screening period and an 8-week treatment period. During the screening period, the patients underwent physical examination, depression and anhedonia scale evaluations, 12-lead ECG, and laboratory examinations.

Patients met the criteria for enrollment after screening were conducted to enter the treatment. Patients were asked to take toludesvenlafaxine hydrochloride sustained-release tablets (Shandong Luye Pharmaceutical Co. Ltd) at a relatively regular time of day, once daily. The recommended dosage was 80 mg to 160 mg per day, starting at 40 mg per day and increasing to 80 mg per day over a week based on individual response, with the maximum dose not exceeding 160 mg per day.

Patients received a total of three visits (end of week 2, end of week 4, and end of week 8) after taking the medication. They received vital signs and weight assessments, depression and anhedonia scale assessments, quality of life and function scale evaluations, adverse effects, and comorbid medication assessments at each visit. Important laboratory tests and a 12-lead ECG were reexamined at the final visit.

### 2.4. Outcomes

#### 2.4.1. Primary Efficacy Indicator

The changes in DARS scale scores from baseline to each visit point in the treatment were recorded. Higher total DARS scores indicate lower levels of anhedonia.

#### 2.4.2. Secondary Efficacy Indicator

Changes in the score of the following scales and related indicators from baseline to every visit of the 8-week treatment are as follows: MADRS total score and MADRS anhedonia factor score; Snaith–Hamilton Pleasure Scale (SHAPS) total score; Sheehan Disability Scale (SDS) total score; and Quality of Life Enjoyment and Satisfaction Questionnaire–Short Form (Q-LES-Q-SF) score. The correlations between the changes from baseline in DARS scores and the changes from baseline in SDS and Q-LES-Q-SF scores were additionally assessed at each follow-up point. The secondary efficacy indicator also included the following: the changes in plasma levels of mBDNF and pro-BDNF, VEGF, and IGF-1 from baseline to the end of treatment; the correlations between the percentage of the changes from baseline in these neurotrophic factors ([8-week-baseline]/baseline); and the percentage of the changes from baseline in DARS, MADRS total score, MADRS anhedonia factor score, SHAPS, SDS, ans Q-LES-Q-SF scores at the week 8 visit ([8-week-baseline]/baseline).

#### 2.4.3. Safety Assessments

Safety assessments carried out at all visits included clinical evaluation of adverse events (AEs) and withdrawal due to AEs, vital signs (temperature, expiration, blood pressure, and pulse), 12-lead ECG, and laboratory tests (hematology, serum chemistry, and urinalysis).

### 2.5. Statistical Analysis

All statistical analyses were conducted using SAS, version 9.4. The level of statistical significance was set at *p*=0.05. Continuous data were presented as mean values along with standard deviations (SDs), while categorical variables were described using frequencies and percentages.

#### 2.5.1. Sample Size

According to a previous study [31], it is assumed that the DARS scale scores of depressed patients with anhedonia increase by at least 10 points at the end of the treatment. On this basis, the sample size was calculated using PASS software, with the following parameters, mean of paired differences = 10, SD of paired differences = 20, power = 0.9, and alpha = 0.005, and the required sample size is estimated to be 63 cases. Considering a conservative drop-out rate of 20%, 79 patients were planned in the trial.

#### 2.5.2. Efficacy Analyses

The efficacy analysis was mainly based on the full-analysis set (FAS), which was defined as all randomized patients who received at least one dose of the study drug during the double-blind treatment period and had both baseline and at least one post-baseline measurement of primary efficacy. The FAS used last-observation carried forward imputation. The differences between the efficacy outcomes of DARS, MARDS, SHAPS, SDS, and Q-LES-Q-SF scores at each follow-up time point and the baseline scores were compared using a paired *t*-test, while the differences in MADRS anhedonia factor score were analyzed using the Wilcoxon signed-rank test due to non-normal distribution. The relationship between the changes in DARS scores from baseline and the changes in SDS and Q-LES-Q-SF at each follow-up point was analyzed using Pearson correlation analysis. The changes in plasma level of pro-BDNF, mBDNF, VEGF, and IGF-1 factors from baseline at the end of treatment were compared using paired *t*-tests. Additionally, Pearson correlation analysis was used to examine the relationships between the percentage of the changes in these factors and the percentage of changes in DARS, MADRS total score, MADRS anhedonia factor score, SHAPS, SDS, and Q-LES-Q-SF scores.

#### 2.5.3. Safety Analysis

Safety analyses were based on the safety population (safety set), which included all patients who received at least 1 dose of the study drug during the treatment period. Participants who discontinued the trial due to AEs and those who experienced serious side effects were listed. The classification, degree of severity, frequency, and relationship with the study drug for all treat-emergent AEs (TEAEs) were summarized. AEs were classified according to Medical Dictionary for Regulatory Activities terminology (MedDRA). All aberrant indicators with clinical relevance were noted, and shift tables were used to describe alterations in the clinically significant assessments of laboratory outcomes.

## 3. Results

### 3.1. Participants

**Figure 1:**
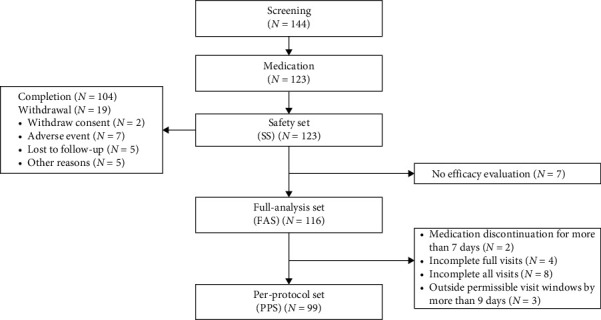
Study design and flow diagram.

From April 2023 to April 2024, a total of 144 participants underwent screening for this study, of which 123 met the enrollment criteria and received medication (safety set). Seven participants did not have at least 1 post-baseline measurement of primary efficacy; thus, 116 participants were included in the FAS set for efficacy analysis. The demographic characteristics of the subjects in the FAS set are presented in Table 1. Among 116 participants, 99 who successfully finished the 8-week treatment with no notable protocol deviation were included in the PPS set (two participants discontinued medication for more than 7 days; four participants did not complete full visits; eight did not complete all visits; and three participants had visits outside the permissible window by more than 9 days). During the course of treatment, 19 individuals discontinued the treatment, and the reasons for discontinuation are as follows: withdraw consent (*N* = 2), AE (*N* = 7), lost to follow-up (*N* = 5), other reasons (*N* = 5) (Figure 1).

### 3.2. Primary Efficacy Indicator

**Figure 2:**
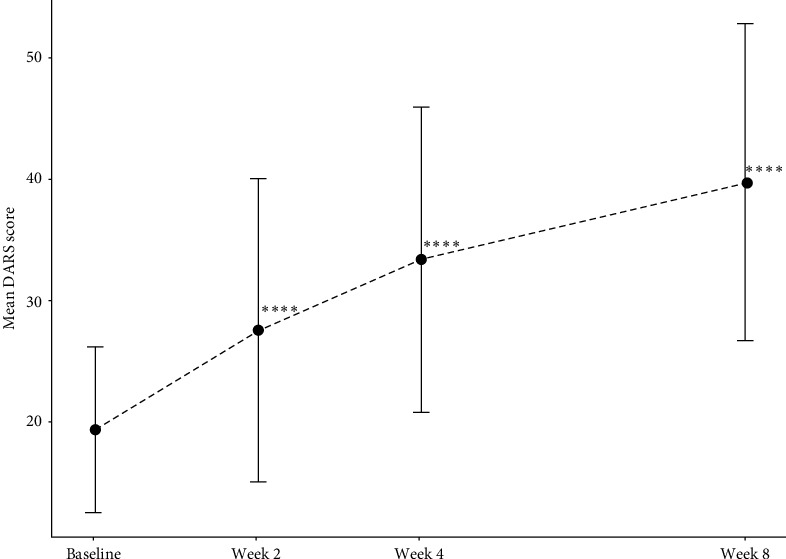
The changes from baseline in DARS total score (FAS). *⁣*^*∗∗∗∗*^ indicates *p* < 0.0001. DARS, Dimensional Anhedonia Rating Scale; FAS, full analysis set.

Table 2 and Figure 2 present the significant improvements in anhedonia across patients over the 8-week treatment (missing data were imputed using LOFT imputation to account for local data patterns and enhance the accuracy of estimates). The mean changes from baseline in DARS scores reached a statistical significance after 2 weeks (mean difference [95% CI]: 8.4 [6.4, 10.4], *p* < 0.0001), 4 weeks (mean difference [95% CI]: 14.1 [12.0,16.2], *p* < 0.0001), and 8 weeks (mean difference [95% CI]: 20.4 [18.0, 22.9], *p* < 0.0001) of treatment.

### 3.3. Secondary Efficacy Indicator

As shown in Table S1/Figure S1 and Table S2/Figure S2, the results were similar for the MADRS total score and MADRS anhedonia factor score, with both scores significantly reduced compared to baseline in patients who received toludesvenlafaxine medication at all follow-up time points (*p* < 0.0001). Particularly, after 8-week treatment, the mean differences to baseline were −22.7 (95% CI: [−24.0, −21.3], *p* < 0.0001) and −13.1 (95% CI: [−13.9,−12.2], *p* < 0.0001]) for MADRS total score and MADRS anhedonia factor score, respectively.

Improvements in anhedonia were also detected by the changes in SHAPS scores. Compared to baseline, SHAPS scores were significantly reduced at every visit after treatment (*p* < 0.0001) (Table S3, Figure S3).

**Figure 3:**
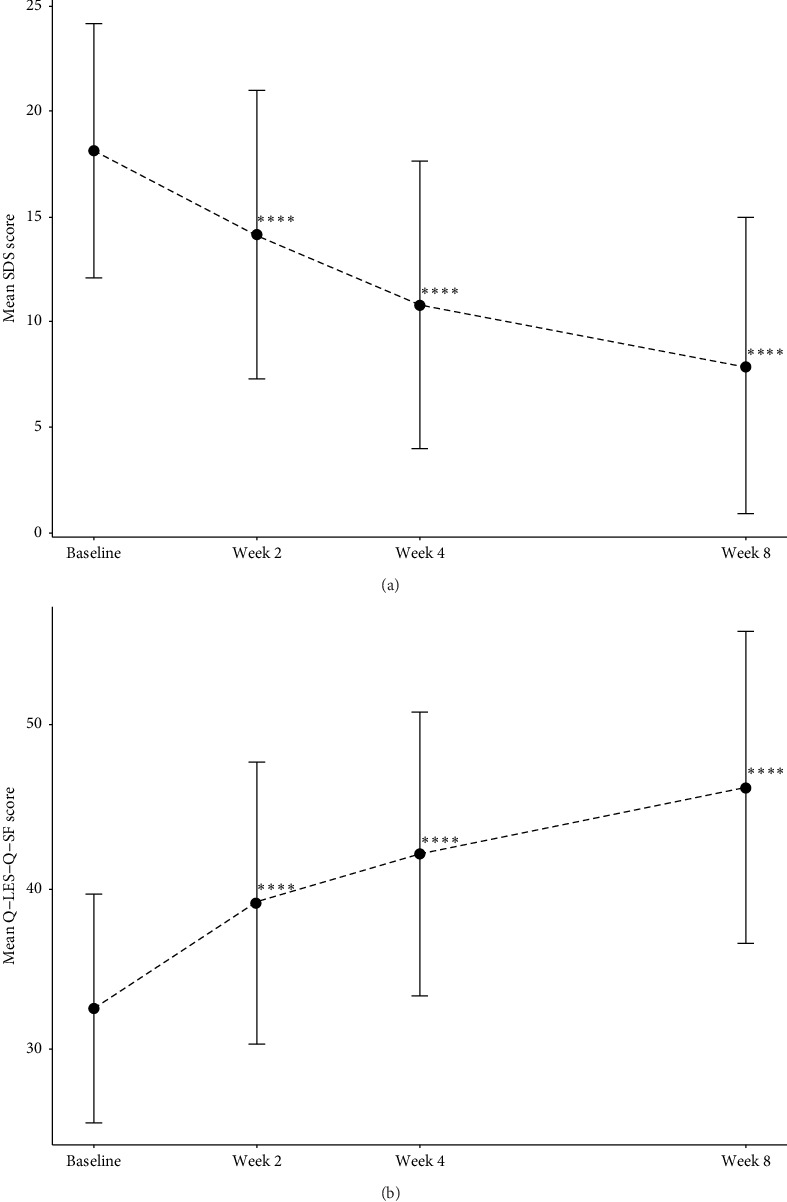
The changes from baseline in SDS total score and in Q-LES-Q-SF total score (FAS). *⁣*^*∗∗∗∗*^ indicates *p* < 0.0001. FAS, full analysis set; Q-LES-Q-SF, Quality of Life Enjoyment and Satisfaction Questionnaire–Short Form; SDS, Sheehan Disability Scale.

A significant lower SDS score emerged after treatment with toludesvenlafaxine, with a significant reduction in scores in comparison to baseline at all three visits (*p* < 0.0001) (Table S4 and Figure 3a); especially, the mean changes from baseline in SDS scores were −10.1 (95% CI: [−11.6, −8.6], *p* < 0.0001) after 8-week treatment. Equivalent results were found for the Q-LES-Q-SF scale; participants who received medication showed a significant boost in Q-LES-Q-SF scores at all following times (*p* < 0.0001) (Table S5 and Figure 3b]), with an increase of 13.9 in Q-LES-Q-SF scores (95% CI: [12.0, 15.9], *p* < 0.0001) at the end of treatment. To assess whether improvements in quality of life and function were associated with the alleviation of anhedonia symptoms, correlation analyses were conducted, as shown in Table 3; the change in DARS score was moderately correlated with a change in SDS score at week 2 (*r* = −0.38, *p* < 0.0001), week 4 (*r* = −0.51, *p* < 0.0001), and week 8 (*r* = −0.49, *p* < 0.0001). Significant positive associations between the change from baseline in Q-LES-Q-SF score and the change in the DARS score were also observed at the follow-up points.

In order to identify the biological markers associated with the improvement of anhedonia symptoms, the change of neurotrophic factors in the plasma was assessed, as shown in Table 4; after 8 weeks of treatment, the levels of mBDNF (*t* = 28.78, *p* < 0.0001), pro-BDNF (*t* = 27.71, *p* < 0.0001), and VEGF (*t* = 31.07, *p* < 0.0001) were significantly increased compared to baseline; the level of IGF-1 was not significantly changed (*t* = 0.35, *p*=0.7269). There was no association between the percentage of changes in these neurotrophic factors and the symptom improvements (Table S6).

### 3.4. Safety

TEAEs were reported in 103 (83.7%, 391 events) of the 123 patients enrolled in the trial; most AEs are mild and moderate in severity. One participant experienced a serious AE (SAE), type 2 diabetes mellitus, which was judged by the investigators to be unrelated to medication use. Six patients (4.9%, 10 events) withdrew from the trial due to adverse effects. Five (4.1%, nine events) out of the six participants discontinued from the study due to the medication. Psychiatric symptoms (two cases) and gastrointestinal system (two cases) symptoms were the main reasons for withdrawal from the trial.

Of 103 participants reporting TEAEs, 94 participants (76.4%, 306 events) were assessed by the investigators as having TRAEs.  Table 5 displayed TRAEs having an incidence ≥5%, arranged by descending frequency for each system. The top three TRAEs were dry mouth, nausea, and dizziness. There were no deaths reported in this study. Detailed information on other safety indicators, including vital signs and laboratory assessments, is presented in Tables S7 and S8.

## 4. Discussion

The purpose of this study was to evaluate the efficacy and safety of toludesvenlafaxine for anhedonia in depressed patients. Overall, the study demonstrated that the TRI drugs significantly improved the symptoms of anhedonia and were well tolerated and safe over the 8-week treatment. This suggests that the toludesvenlafaxine may be a potential therapy for anhedonia in depression.

We observed that the primary outcome DARS scores increased significantly after 2 weeks of treatment, with scores approaching clinical remission levels (Figure 2) and improved steadily over the 8-week trial. Consistent with previous findings [29], these results suggest that toludesvenlafaxine is capable of alleviating symptoms of anhedonia in depression. Moreover, in contrast with conventional antidepressants, toludesvenlafaxine demonstrated more rapid efficacy in treating anhedonia, which often requires 4–8 weeks to achieve comparable effects on anhedonia [32, 33]. For instance, Dudek et al. [33] found that anhedonia symptoms reached remission after 8 weeks of SSRI medication and achieved anhedonia relief after 4 weeks of trazodone treatment. These data implicated the superior efficacy of toludesvenlafaxine in targeting DA pathways critical for reward processing compared with conventional antidepressants. Unlike a recent study that only used the MADRS anhedonia factor score to assess anhedonia symptoms, we used the second-generation scales, DARS to specifically probe the different anhedonia subdomains, including anticipatory versus consummatory anhedonia, which allowed for more accurate results in the current study [34]. Notably, toludesvenlafaxine improved DARS at 4 weeks of treatment (an average improvement of 14.1 points compared with baseline) to a degree comparable to that of 8-week pramipexole (DA receptor agonist) [31] conjunctive medication, further demonstrating the superiority of TRIs drugs for the treatment of anhedonia.

Reinforced by the results of the MADRS anhedonia factor score and SHAPS total score, we similarly found a significant amelioration of anhedonia by toludesvenlafaxine.

Utilizing MADRS total scores, we assessed the efficacy of the treatment of depressive symptoms and found a significant effect of toludesvenlafaxine on the improvement of depressive symptoms, which replicates the results of previous studies [29, 35]. In addition, the antidepressant effect of toludesvenlafaxine was more potent compared to other SSRIs and SNRIs drugs; for instance, the MADRS subtraction scores of 8 weeks of treatment with 120 mg/d duloxetine or 20 mg/day paroxetine was −14.1 and −13.8, respectively [36], which may be moderate compared to −22.7 for the drug used in our study. These results add strong evidence for the efficacy of toludesvenlafaxine in depression.

An important aspect of the treatment of depressive disorders is the restoration of function and the improvement of quality of life [37]. In the present study, we found that toludesvenlafaxine significantly reduced the SDS total score (mean changes of −10.1 points compared to baseline at week 8). Parallelly, toludesvenlafaxine significantly increased the Q-LES-Q-SF total score (mean changes of +13.9 points at week 8) and improved patients' subjective satisfaction with life. Therefore, we confirmed, in two dimensions, the enhancing effect of toludesvenlafaxine on the overall status of patients and that this ameliorative effect of 8-week toludesvenlafaxine was also even stronger than that of the 9-week agomelatine (changes of −8.43 points compared to baseline for SDS and changes of +9.47 points compared to baseline for Q-LES-Q-SF after 9-week agomelatine medication) [38, 39]. Coinciding with a previous study [17], we also found that the improvements in functioning and quality of life were significantly associated with improvements in anhedonia (Table 3), rendering the importance of anhedonia management in MDDs [6].

To further explore how toludesvenlafaxine functions in improving anhedonia, we measured the level of BDNF, VEGF, and IGF-1 after the 8 weeks of treatment. We observed that the toludesvenlafaxine significantly increased BDNF and VEGF levels; however, this increase did not correlate with the improvement of anhedonia. This finding suggests that neurotrophic factors may not be a direct driver of anhedonia alleviation but rather a byproduct of the treatment process. Previous studies indicated that DA medications could enhance neuroplasticity, thereby influencing mood states [40]. Notably, while DA medications can enhance neuroplasticity (as indicated by increases in BDNF and VEGF), the improvement in anhedonia and depressive symptoms likely requires further general rehabilitation of reward and other related brain circuits [41]. Consistent with previous studies [42], 8 weeks of toludesvenlafaxine medication may be not long enough to observe a significant correlation between the changes in neurotrophic factors and symptom improvements. The mechanisms underlying the three-channel drug's effects may be more complex, involving multiple neurotransmitter interactions. Additionally, anhedonia may be influenced by other biological markers, such as the endogenous opioid system [43]. Furthermore, peripheral plasma levels of neurotrophic factors may not fully capture their dynamic roles in specific brain regions (e.g., amygdala or subgenual ACC), where localized mRNA expression of BDNF-dependent genes could differentially distinguish depression or anhedonia subdomains [44]. Our results also showed that the plasma level of IGF-1 was not significantly altered following treatment. However, this does not necessarily imply that IGF-1 is unrelated to anhedonia or the treatment. Previous studies have indicated that depressed patients do not consistently exhibit higher levels of IGF-1 compared to healthy individuals [45]. Therefore, the treatment may have corrected abnormal IGF-1 levels in some individuals, but this did not result in a significant change at the group level. These highlight the need for further research to elucidate the potential mechanisms linking neurotrophic factors and anhedonia.

In our study, we found that toludesvenlafaxine medication was safe and well-tolerated. The TEAEs were mild to moderated and no serious adverse effects occurred due to the medication. In the current study, 4.1% of participants withdrew from the trial due to the adverse effects related to toludesvenlafaxine.. The drop rates attributed to adverse effects were 5.3%–5.4% for escitalopram, 6.8% for vortioxetine, and 12.9% for SNRIs (duloxetine and venlafaxine) [46, 47], illustrating the superior safety and tolerability of toludesvenlafaxine compared to other common antidepressants. The most common drug-related adverse effects include dry mouth, nausea, constipation, diarrhea, vomiting, dizziness, headache, and drowsiness, which are similar to the common adverse effects of SSRIs and SNRIs [48, 49].

Sexual dysfunction (SD) is one of the most common adverse effects of antidepressants, resulting in premature discontinuation of antidepressant treatment, relapse, and worsened health outcomes and quality [50]. Studies have reported that the prevalence of treatment-emergent SD in depressed patients prescribed either an SSRI or SNRI to be between 37.1 and 61.5% [51, 52], whereas the incidence of SD in our study was only 1%, further demonstrating the ability of toludesvenlafaxine to ameliorate anhedonia and depressive symptoms with less impact on the patient's quality of life.

These findings underscore the potential clinical significance of toludesvenlafaxine as a promising therapeutic method for improving both depressive symptoms and quality of life in patients with significant anhedonia. Especially, toludesvenlafaxine demonstrated a more rapid onset of action, with significant improvements in anhedonia observed as early as 2 weeks of treatment compared with other antidepressants. This quick response may offer patients faster relief, a crucial factor in managing depression. Moreover, compared to other antidepressants, toludesvenlafaxine has a much lower incidence of side effects, especially sexual side effects. This suggests that toludesvenlafaxine may be a safer option for patients with depression and could serve as an alternative for those who experience SD from other antidepressants.

This study has certain limitations. First, it lacked a control group, making it difficult to rule out potential confounding factors. However, based on phase III clinical trials, the placebo effect in the treatment of anhedonia was modest [29]. Secondly, the study only evaluated the improvement in anhedonia by subjective rating scales and did not perform objective behavioral tasks for assessing anhedonia, such as the Probabilistic Reward Task [53]. Thirdly, the lack of a control group may affect the interpretability of the results. Incorporating a control group, such as a placebo or an existing standard treatment, in future research would provide a more comprehensive evaluation of toludesvenlafaxine's efficacy and safety. Finally, the sample size of this study was small. Future studies recruiting more participants are needed to confirm the efficacy and safety of toludesvenlafaxine.

Still, to our best knowledge, the study is the first to use anhedonia-specific scales to establish the efficacy and safety of toludesvenlafaxine in the treatment of anhedonia and brings avenues to improve the overall quality of life in patients with depression.

## Figures and Tables

**Table 1:** Demographic and baseline characteristics (FAS).

	Toludesvenlafaxine hydrochloride sustained-release tablets (*n* = 116)
Age (year)	—
Mean ± SD	28.2 ± 9.0
Sex, *n* (%)	—
Male	40 (34.5)
Female	75 (64.7)
Han, *n* (%)	111 (95.7)
Weight (kg)	—
Mean ± SD	64.20 ± 15.08
DARS total score	—
Mean ± SD	19.3 ± 6.9
MADRS total score	—
Mean ± SD	32.4 ± 4.1
SHAPS total score	—
Mean ± SD	36.3 ± 5.5
SDS total score	—
Mean ± SD	18.1 ± 6.0
Q-LES-Q-SF total score	—
Mean ± SD	32.5 ± 7.0

Abbreviations: DARS, Dimensional Anhedonia Rating Scale; FAS, full-analysis set; MADRS, Montgomery–Asberg Depression Rating Scale; Q-LES-Q-SF, Quality of Life Enjoyment and Satisfaction Questionnaire–Short Form; SDS, Sheehan Disability Scale; SHAPS, Snaith–Hamilton Pleasure Scale.

**Table 2:** The changes from baseline in DARS total score (FAS).

	Toludesvenlafaxine hydrochloride sustained-release tablets (*n* = 116)
Baseline	—
*N* (missing)	116 (0)
Mean ± SD	19.3 ± 6.9
2-week treatment	—
*N* (missing)	116 (0)
Mean ± SD	27.6 ± 12.4
2-week treatment—baseline	—
N (missing)	116 (0)
Mean ± SD	8.4 ± 10.9
Mean (95% CI)	(6.4, 10.4)
* t*	8.27
* p*	<0.0001
4-week treatment	—
*N* (missing)	116 (0)
Mean ± SD	33.4 ± 12.6
4-week treatment—baseline	—
*N* (missing)	116 (0)
Mean ± SD	14.1 ± 11.5
Mean (95% CI)	(12.0, 16.2)
* t*	13.20
* p*	<0.0001
8-week treatment	—
*N* (missing)	116 (0)
Mean ± SD	39.7 ± 13.0
8-week treatment—baseline	—
*N* (missing)	116 (0)
Mean ± SD	20.4 ± 13.2
Mean (95% CI)	(18.0, 22.9)
* t*	16.63
* p*	<0.0001

*Note*: The DARS scale score loss was filled in by the LOCF method. Comparisons of change from baseline in treatment xx weeks were performed using paired *t*-test.

**Table 3:** Correlation analysis of DARS score change and SDS and Q-LES-Q-SF score improvement (FAS).

	Toludesvenlafaxine hydrochloride sustained-release tablets		
	DARS total score		
	2-week treatment—baseline	4-week treatment—baseline	8-week treatment—baseline
SDS total score	—	—	—
2-week treatment—baseline	*n* = 115 (*r* = −0.38237) (*p* < 0.0001)	—	—
4-week treatment—baseline	—	*n* = 108 (*r* = −0.50512) (*p* < 0.0001)	—
8-week treatment—baseline	—	—	*n* = 104 (*r* = −0.48748) (*p* < 0.0001)
Q-LES-Q-SF total score	—	—	—
2-week treatment—baseline	*n* = 115 (*r* = 0.43401) (*p* < 0.0001)	—	—
4-week treatment—baseline	—	*n* = 108 (*r* = 0.49270) (*p* < 0.0001)	—
8-week treatment—baseline	—	—	*n* = 104 (*r* = 0.46176) (*p* < 0.0001)

*Note*: Pearson correlation analysis was used to calculate the paired samples, correlation coefficients, and *p*-value.

**Table 4:** The changes from baseline in neurotrophic factors in the plasma (FAS).

		Toludesvenlafaxine hydrochloride sustained-release tablets (*n* = 116)
mBDNF	—	—
Baseline	*N* (Nmiss)	104 (12)
	Mean ± SD	1248.856 ± 305.634
8-week treatment	*N* (Nmiss)	104 (12)
	Mean ± SD	2420.882 ± 263.793
8-week treatment—baseline	*N* (Nmiss)	104 (12)
	Mean ± SD	1172.026 ± 415.351
	Mean (95%CI)	(1091.251, 1252.801)
	*t*	28.78
	*p*	<0.0001
pro-BDNF	—	—
Baseline	*N* (Nmiss)	104 (12)
	Mean ± SD	618.059 ± 141.249
8-week treatment	*N* (Nmiss)	104 (12)
	Mean ± SD	1160.575 ± 137.704
8-week treatment—baseline	*N* (Nmiss)	104 (12)
	Mean ± SD	542.516 ± 199.645
	Mean (95%CI)	(503.690, 581.342)
	*t*	27.71
	*p*	<0.0001
VEGF	—	—
Baseline	*N* (Nmiss)	104 (12)
	Mean ± SD	176.647 ± 34.953
8-week treatment	*N* (Nmiss)	104 (12)
	Mean ± SD	314.135 ± 34.916
8-week treatment—baseline	*N* (Nmiss)	104 (12)
	Mean ± SD	137.488 ± 45.122
	Mean (95%CI)	(128.713, 146.263)
	*t*	31.07
	*p*	<0.0001
IGF-1	—	—
Baseline	*N* (Nmiss)	104(12)
	Mean ± SD	753.662 ± 183.658
8-week treatment	N (Nmiss)	104 (12)
	Mean ± SD	744.336 ± 197.103
8-week treatment—baseline	*N* (Nmiss)	104 (12)
	Mean ± SD	−9.326 ± 271.591
	Mean (95%CI)	(−62.144, 43.491)
	*t*	0.35
	*p*	0.7269

*Note*: Comparisons of change from baseline in treatment xx weeks were performed using paired *t*-test.

**Table 5:** Treatment-related adverse events with an incidence ≥5% in treatment group (SS).

SOC (PT)	Toludesvenlafaxine hydrochloride sustained-release tablets *n* (%)
Treatment-related adverse events	94 (76.4)
Gastrointestinal disorders	69 (56.1)
Dry mouth	34 (27.6)
Nausea	27 (22.0)
Constipation	16 (13.0)
Vomiting	9 (7.3)
Diarrhea	9 (7.3)
Epigastric pain	1 (0.8)
Hygrostomia	1 (0.8)
Belching	1 (0.8)
Dysemesia	1 (0.8)
Abdominal discomfort	1 (0.8)
Nervous system disorders	47 (38.2)
Dizziness	26 (21.1)
Drowsiness	16 (13.0)
Headache	14 (11.4)
Fremitus	4 (3.3)
Sleepy	3 (2.4)
Parageusia	2 (1.6)
Head discomfort	1 (0.8)
Paresthesia	1 (0.8)
Bradykinesia	1 (0.8)
Cathisophobia	1 (0.8)
Metabolism and nutrition disorders	21 (17.1)
Decreased appetite	21 (17.1)
Psychiatric disorders	20 (16.3)
Insomnia	16 (13.0)
Dyssomnia	2 (1.6)
Decreased libido	1 (0.8)
Depressed	1 (0.8)
Irritability	1 (0.8)
Anxious	1 (0.8)
Skin and subcutaneous tissue disorders	16 (13.0)
Hyperhidrosis	11 (8.9)
Pruritus	3 (2.4)
Papula	1 (0.8)
Photosensitivity reaction	1 (0.8)
Night sweat	1 (0.8)
Red rash	1 (0.8)
Eye organ disorders	12 (9.8)
Visual blur	12 (9.8)
Investigations	10 (8.1)
Weight loss	6 (4.9)
Decreased blood pressure	2(1.6)
Aspartate aminotransferase increased	1 (0.8)
Aspartate transaminase increased	1 (0.8)
Increased blood pressure	1 (0.8)
Bilirubin increased	1 (0.8)
Systemic disease and reactions of administration site	6 (4.9)
Chest discomfort	3 (2.4)
Activity reduction	2 (1.6)
Peripheral swelling	1 (0.8)
Cardiac disorders	5 (4.1)
Palpitations	4 (3.3)
Sychnosphygmia	1 (0.8)
Respiratory, thoracic, and mediastinal disorders	4 (3.3)
Rhinobyon	3 (2.4)
Epis	1 (0.8)
Musculoskeletal and connective tissue disorders	2 (1.6)
Myalgia	2 (1.6)
Body pain	1 (0.8)
Immune system disorders	1 (0.8)
Hypersensitivity	1 (0.8)
Injury, poisoning, and operational complications	1 (0.8)
Heatstroke	1 (0.8)
Infection and infestation disorders	1 (0.8)
Upper respiratory infection	1 (0.8)
Reproductive system and breast disorders	1 (0.8)
Sexual dysfunction	1 (0.8)
Ear and labyrinth disorders	1 (0.8)
Giddiness	1 (0.8)
Kidney and urinary system disorders	1 (0.8)
Hematuria	1 (0.8)

*Note*: There were 123 patients in the group.

Abbreviations: PT, preferred term; SOC, system organ class.

## Data Availability

The data and statistical analysis plan used in this study will be made available upon reasonable request after publication. To access the data, please contact the corresponding author via email.

## Supporting Information

Supporting Information 1 Table S1. The changes from baseline in MADRS total score (FAS). Table S2. The changes from baseline in MADRS anhedonia factor score (FAS). Table S3. The changes from baseline in SHAPS total score (FAS). Table S4. The changes from baseline in SDS total score (FAS). Table S5. The changes from baseline in Q-LES-Q-SF total score (FAS). Table S6. Correlation analysis of neurotrophic factors and symptom improvements (FAS). Table S7. The changes from baseline in mean supine systolic and diastolic pressure. Table S8. Treatment-emergent adverse events of laboratory tests.

Supporting Information 2 Figure S1. The changes from baseline in MADRS total score (FAS). *⁣*^*∗*^*⁣*^*∗*^*⁣*^*∗*^*⁣*^*∗*^ indicates *p* < 0.0001. FAS, full analysis set; MADRS, Montgomery–Asberg Depression Rating Scale.

Supporting Information 3 Figure S2. Changes from baseline in MADRS anhedonia factor score (FAS). *⁣*^*∗*^*⁣*^*∗*^*⁣*^*∗*^*⁣*^*∗*^ indicates *p* < 0.0001. FAS, full analysis set; MADRS, Montgomery–Asberg Depression Rating Scale.

Supporting Information 4 Figure S3. Changes from baseline in SHAPS score (FAS). *⁣*^*∗*^*⁣*^*∗*^*⁣*^*∗*^*⁣*^*∗*^ indicates *p* < 0.0001. FAS, full analysis set; SHAPS, Snaith–Hamilton Pleasure Scale.

## References

[1] Herrman H., Kieling C., McGorry P., Horton R., Sargent J., Patel V. (2019). Reducing the Global Burden of Depression: A Lancet-World Psychiatric Association Commission. *Lancet*.

[2] Malhi G. S., Mann J. J. (2018). Depression.. *Lancet*.

[3] Der-Avakian A., Markou A. (2012). The Neurobiology of Anhedonia and Other Reward-Related Deficits. *Trends in Neurosciences*.

[4] Goodyer I. M., Reynolds S., Barrett B. (2017). Cognitive Behavioural Therapy and Short-Term Psychoanalytical Psychotherapy Versus a Brief Psychosocial Intervention in Adolescents With Unipolar Major Depressive Disorder (IMPACT): A Multicentre, Pragmatic, Observer-Blind, Randomised Controlled Superiority Trial. *Lancet Psychiatry*.

[5] Ducasse D., Loas G., Dassa D. (2018). Anhedonia Is Associated With Suicidal Ideation Independently of Depression: A Meta-Analysis. *Depression and Anxiety*.

[6] Costi S., Morris L. S., Kirkwood K. A. (2021). Impact of the KCNQ2/3 Channel Opener Ezogabine on Reward Circuit Activity and Clinical Symptoms in Depression: Results From a Randomized Controlled Trial. *American Journal of Psychiatry*.

[7] Pizzagalli D. A. (2022). Toward a Better Understanding of the Mechanisms and Pathophysiology of Anhedonia: Are We Ready for Translation?. *American Journal of Psychiatry*.

[8] Sanacora G., Yan Z., Popoli M. (2022). The Stressed Synapse 2.0: Pathophysiological Mechanisms in Stress-Related Neuropsychiatric Disorders. *Nature Reviews Neuroscience*.

[9] Berridge K. C., Kringelbach M. L. (2015). Pleasure Systems in the Brain. *Neuron*.

[10] Coenen V. A., Schlaepfer T. E., Maedler B., Panksepp J. (2011). Cross-Species Affective Functions of the Medial Forebrain Bundle-Implications for the Treatment of Affective Pain and Depression in Humans. *Neuroscience & Biobehavioral Reviews*.

[11] Bracht T., Doidge A. N., Keedwell P. A., Jones D. K. (2015). Hedonic Tone Is Associated With Left Supero-Lateral Medial Forebrain Bundle Microstructure. *Psychological Medicine*.

[12] Chaudhury D., Walsh J. J., Friedman A. K. (2013). Rapid Regulation of Depression-Related Behaviours by Control of Midbrain Dopamine Neurons. *Nature*.

[13] Willmore L., Cameron C., Yang J., Witten I. B., Falkner A. L. (2022). Behavioural and Dopaminergic Signatures of Resilience. *Nature*.

[14] Wang H.-Q., Wang Z.-Z., Chen N.-H. (2021). The Receptor Hypothesis and the Pathogenesis of Depression: Genetic Bases and Biological Correlates. *Pharmacological Research*.

[15] Ren H., Fabbri C., Uher R. (2018). Genes Associated With Anhedonia: A New Analysis in a Large Clinical Trial (GENDEP). *Translational Psychiatry*.

[16] Noma H., Furukawa T. A., Maruo K. (2019). Exploratory Analyses of Effect Modifiers in the Antidepressant Treatment of Major Depression: Individual-Participant Data Meta-Analysis of 2803 Participants in Seven Placebo-Controlled Randomized Trials. *Journal of Affective Disorders*.

[17] McIntyre R. S., Necking O., Schmidt S. N., Reines E. (2024). Minimal Clinically Important Change in the MADRS Anhedonia Factor Score: A Pooled Analysis of Open-Label Studies With Vortioxetine in Patients With Major Depressive Disorder. *Journal of Affective Disorders*.

[18] Serretti A. (2023). Anhedonia and Depressive Disorders. *Clinical Psychopharmacology and Neuroscience*.

[19] Kennedy S. H., Lam R. W., Rotzinger S. (2019). Symptomatic and Functional Outcomes and Early Prediction of Response to Escitalopram Monotherapy and Sequential Adjunctive Aripiprazole Therapy in Patients With Major Depressive Disorder: A CAN-BIND-1 Report. *The Journal of Clinical Psychiatry*.

[20] Kotan Z., Sarandöl E., Kırhan E., Ozkaya G., Kırlı S. (2012). Serum Brain-Derived Neurotrophic Factor, Vascular Endothelial Growth Factor and Leptin Levels in Patients With a Diagnosis of Severe Major Depressive Disorder With Melancholic Features. *Therapeutic Advances in Psychopharmacology*.

[21] Wu C., Lu J., Lu S., Huang M., Xu Y. (2020). Increased Ratio of Mature BDNF to Precursor-BDNF in Patients With Major Depressive Disorder With Severe Anhedonia. *Journal of Psychiatric Research*.

[22] Kopczak A., Stalla G. K., Uhr M. (2015). IGF-I in Major Depression and Antidepressant Treatment Response. *European Neuropsychopharmacology*.

[23] Arinami H., Watanabe Y., Suzuki Y., Tajiri M., Tsuneyama N., Someya T. (2023). Serum Cortisol and Insulin-Like Growth Factor 1 Levels in Major Depressive Disorder and Schizophrenia. *Scientific Reports*.

[24] Bustos G., Abarca J., Campusano J., Bustos V., Noriega V., Aliaga E. (2004). Functional Interactions Between Somatodendritic Dopamine Release, Glutamate Receptors and Brain-Derived Neurotrophic Factor Expression in Mesencephalic Structures of the Brain. *Brain Research Reviews*.

[25] Pristerà A., Blomeley C., Lopes E. (2019). Dopamine Neuron-Derived IGF-1 Controls Dopamine Neuron Firing, Skill Learning, and Exploration. *Proceedings of the National Academy of Sciences*.

[26] Vaishnavi S. N., Nemeroff C. B., Plott S. J., Rao S. G., Kranzler J., Owens M. J. (2004). Milnacipran: A Comparative Analysis of Human Monoamine Uptake and Transporter Binding Affinity. *Biological Psychiatry*.

[27] Zhu H., Wang W., Sha C. (2021). Pharmacological Characterization of Toludesvenlafaxine as a Triple Reuptake Inhibitor. *Frontiers in Pharmacology*.

[28] Zhang R., Li X., Shi Y. (2014). The Effects of LPM570065, a Novel Triple Reuptake Inhibitor, on Extracellular Serotonin, Dopamine and Norepinephrine Levels in Rats. *PLoS ONE*.

[29] Mi W., Di X., Wang Y. (2023). A Phase 3, Multicenter, Double-Blind, Randomized, Placebo-Controlled Clinical Trial to Verify the Efficacy and Safety of Ansofaxine (LY03005) for Major Depressive Disorder. *Translational Psychiatry*.

[30] Lin J., Su Y., Rizvi S. J. (2022). Define and Characterize the Anhedonia in Major Depressive Disorder: An Explorative Study. *Journal of Affective Disorders*.

[31] Ventorp F., Lindahl J., van Westen D., Jensen J., Björkstrand J., Lindqvist D. (2022). Preliminary Evidence of Efficacy and Target Engagement of Pramipexole in Anhedonic Depression. *Psychiatric Research and Clinical Practice*.

[32] McIntyre R. S., Agid O., Biesheuvel E., Purushottamahanti P. (2024). Effect of Venlafaxine on Anhedonia and Amotivation in Patients With Major Depressive Disorder. *CNS Spectrums*.

[33] Dudek D., Chrobak A. A., Krupa A. J. (2023). TED-Trazodone Effectiveness in Depression: A Naturalistic Study of the Effeciveness of Trazodone in Extended Release Formulation Compared to SSRIs in Patients With a Major Depressive Disorder. *Frontiers in Pharmacology*.

[34] Rizvi S. J., Quilty L. C., Sproule B. A., Cyriac A., Michael Bagby R., Kennedy S. H. (2015). Development and Validation of the Dimensional Anhedonia Rating Scale (DARS) in a Community Sample and Individuals With Major Depression. *Psychiatry Research*.

[35] Mi W., Yang F., Li H. (2022). Safety, and Tolerability of Ansofaxine (LY03005) Extended-Release Tablet for Major Depressive Disorder: A Randomized, Double-Blind, Placebo-Controlled, Dose-Finding, Phase 2 Clinical Trial. *International Journal of Neuropsychopharmacology*.

[36] Detke M. J., Wiltse C. G., Mallinckrodt C. H., McNamara R. K., Demitrack M. A., Bitter I. (2004). Duloxetine in the Acute and Long-Term Treatment of Major Depressive Disorder: A Placebo- and Paroxetine-Controlled Trial. *European Neuropsychopharmacology*.

[37] Marwaha S., Palmer E., Suppes T., Cons E., Young A. H., Upthegrove R. (2023). Novel and Emerging Treatments for Major Depression. *The Lancet*.

[38] Huang J., Xie X.-M., Lyu N. (2023). Agomelatine in the Treatment of Anhedonia, Somatic Symptoms, and Sexual Dysfunction in Major Depressive Disorder. *Frontiers in Psychiatry*.

[39] Di Giannantonio M., Martinotti G. (2012). Anhedonia and Major Depression: The Role of Agomelatine. *European Neuropsychopharmacology*.

[40] Duman R. S., Aghajanian G. K. (2012). Synaptic Dysfunction in Depression: Potential Therapeutic Targets. *Science*.

[41] Casarotto P. C., Girych M., Fred S. M. (2021). Antidepressant Drugs Act by Directly Binding to TRKB Neurotrophin Receptors. *Cell*.

[42] Gelle T., Samey R. A., Plansont B. (2021). BDNF and pro-BDNF in Serum and Exosomes in Major Depression: Evolution After Antidepressant Treatment. *Progress in Neuro-Psychopharmacology and Biological Psychiatry*.

[43] Koob G. F., Volkow N. D. (2016). Neurobiology of Addiction: A Neurocircuitry Analysis. *The Lancet Psychiatry*.

[44] Tripp A., Oh H., Guilloux J.-P., Martinowich K., Lewis D. A., Sibille E. (2012). Brain-Derived Neurotrophic Factor Signaling and Subgenual Anterior Cingulate Cortex Dysfunction in Major Depressive Disorder. *American Journal of Psychiatry*.

[45] Sharma A. N., da Costa, Silva B. F. B., Soares J. C., Carvalho A. F., Quevedo J. (2016). Role of Trophic Factors GDNF, IGF-1 and VEGF in Major Depressive Disorder: A Comprehensive Review of Human Studies. *Journal of Affective Disorders*.

[46] Kennedy S. H., Andersen H. F., Thase M. E. (2009). Escitalopram in the Treatment of Major Depressive Disorder: A Meta-Analysis. *Current Medical Research and Opinion*.

[47] Boulenger J.-P., Loft H., Olsen C. K. (2014). Efficacy and Safety of Vortioxetine (Lu AA21004), 15 and 20 mg/Day:A Randomized, Double-Blind, Placebo-Controlled, Duloxetine-Referenced Study in the Acute Treatment of Adult Patients With Major Depressive Disorder. *International Clinical Psychopharmacology*.

[48] Cipriani A., Santilli C., Furukawa T. A. (2016). Escitalopram Versus Other Antidepressive Agents for Depression. *Cochrane Database of Systematic Reviews*.

[49] Cipriani A., Koesters M., Furukawa T. A. (2012). Duloxetine Versus Other Anti-Depressive Agents for Depression. *Cochrane Database of Systematic Reviews*.

[50] Kishi T., Ikuta T., Sakuma K. (2023). Antidepressants for the Treatment of Adults With Major Depressive Disorder in the Maintenance Phase: A Systematic Review and Network Meta-Analysis. *Molecular Psychiatry*.

[51] Reichenpfader U., Gartlehner G., Morgan L. C. (2014). Sexual Dysfunction Associated With Second-Generation Antidepressants in Patients With Major Depressive Disorder: Results From a Systematic Review With Network Meta-Analysis. *Drug Safety*.

[52] Pillinger T., Howes O. D., Correll C. U. (2023). Antidepressant and Antipsychotic Side-Effects and Personalised Prescribing: A Systematic Review and Digital Tool Development. *The Lancet Psychiatry*.

[53] Krystal A. D., Pizzagalli D. A., Smoski M. (2020). A Randomized Proof-of-Mechanism Trial Applying the Fast-Fail Approach to Evaluating *κ*-Opioid Antagonism as a Treatment for Anhedonia. *Nature Medicine*.

