# Supplementary material for: Efficacy and Safety of Toludesvenlafaxine Hydrochloride Sustained-Release Tablets in Depression With Anhedonia: A Single-Arm, Multicenter Clinical Study
Source: Depress Anxiety. 2025 May 5;2025:6130764. doi: 10.1155/da/6130764 (PMC12069848; doi:10.1155/da/6130764)
Supplement: Supporting Information 1 — Table S1. The changes from baseline in MADRS total score (FAS). Table S2. The changes from baseline in MADRS anhedonia factor score (FAS). Table S3. The changes from baseline in SHAPS total score (FAS). Table S4. The changes from baseline in SDS total score (FAS). Table S5. The changes from baseline in Q-LES-Q-SF total score (FAS). Table S6. Correlation analysis of neurotrophic factors and symptom improvements (FAS). Table S7. The changes from baseline in mean supine systolic and diastolic pressure. Table S8. Treatment-emergent adverse events of laboratory tests. [file 6130764.f1.docx]

**Supplementary Tables**

Supplementary Table 1 The Changes from Baseline in MADRS Total Score (FAS)

|  | **Toludesvenlafaxine hydrochloride sustained-release tablets**  **(n=116)** |
| --- | --- |
| Baseline |  |
| N (Missing) | 116(0) |
| Mean±SD | 32.4±4.1 |
| 2-week treatment |  |
| N (Missing) | 115(1) |
| Mean±SD | 21.9±7.3 |
| 2-week treatment -Baseline |  |
| N (Missing) | 115(1) |
| Mean±SD | -10.5±6.8 |
| Mean(95%CI) | (-11.7,-9.2) |
| *t* | 16.65 |
| *P* | <0.0001 |
| 4-week treatment |  |
| N (Missing) | 108(8) |
| Mean±SD | 15.3±7.4 |
| 4-week treatment -Baseline |  |
| N (Missing) | 108(8) |
| Mean±SD | -17.0±7.0 |
| Mean(95%CI) | (-18.3,-15.6) |
| *t* | 25.04 |
| *P* | <0.0001 |
| 8-week treatment |  |
| N (Missing) | 105(11) |
| Mean±SD | 9.7±7.0 |
| 8-week treatment -Baseline |  |
| N (Missing) | 105(11) |
| Mean±SD | -22.7±6.9 |
| Mean(95%CI) | (-24.0,-21.3) |
| *t* | 33.55 |
| *P* | <0.0001 |

Comparisons of change from baseline in treatment xx weeks were performed using paired t test.

Supplementary Table 2 The Changes from Baseline in MADRS anhedonia factor score (FAS)

|  | **Toludesvenlafaxine hydrochloride sustained-release tablets**  **(n=116)** |
| --- | --- |
| Baseline |  |
| N (Missing) | 116(0) |
| Mean±SD | 18.8±2.4 |
| 2-week treatment |  |
| N (Missing) | 115(1) |
| Mean±SD | 12.8±4.3 |
| 2-week treatment -Baseline |  |
| N (Missing) | 115(1) |
| Mean±SD | -6.1±4.2 |
| Mean(95%CI) | (-6.8,-5.3) |
| *S* | 3091.00 |
| *P* | <0.0001 |
| 4-week treatment |  |
| N (Missing) | 108(8) |
| Mean±SD | 9.0±4.4 |
| 4-week treatment -Baseline |  |
| N (Missing) | 108(8) |
| Mean±SD | -9.8±4.5 |
| Mean(95%CI) | (-10.6,-8.9) |
| *S* | 2884.00 |
| *P* | <0.0001 |
| 8-week treatment |  |
| N (Missing) | 105(11) |
| Mean±SD | 5.8±4.2 |
| 8-week treatment -Baseline |  |
| N (Missing) | 105(11) |
| Mean±SD | -13.1±4.3 |
| Mean(95%CI) | (-13.9,-12.2) |
| *S* | 2782.50 |
| *P* | <0.0001 |

Anhedonia factors include: item 1 (external sadness), 2 (sad experience), 6 (difficulty concentrating), 7 (burnout), and 8 (not feeling).

Comparisons of change from baseline in treatment xx weeks were performed using Wilcoxon signed-rank test.

Supplementary Table 3 The Changes from Baseline in SHAPS Total Score (FAS)

|  | **Toludesvenlafaxine hydrochloride sustained-release tablets**  **(n=116)** |
| --- | --- |
| Baseline |  |
| N (Missing) | 115(1) |
| Mean±SD | 36.3±5.5 |
| 2-week treatment |  |
| N (Missing) | 115(1) |
| Mean±SD | 33.8±5.9 |
| 2-week treatment -Baseline |  |
| N (Missing) | 115(1) |
| Mean±SD | -2.5±5.2 |
| Mean(95%CI) | (-3.4,-1.5) |
| *t* | 5.03 |
| *P* | <0.0001 |
| 4-week treatment |  |
| N (Missing) | 108(8) |
| Mean±SD | 31.3±6.1 |
| 4-week treatment -Baseline |  |
| N (Missing) | 108(8) |
| Mean±SD | -5.4±5.4 |
| Mean(95%CI) | (-6.4,-4.3) |
| *t* | 10.27 |
| *P* | <0.0001 |
| 8-week treatment |  |
| N (Missing) | 105(11) |
| Mean±SD | 28.8±7.0 |
| 8-week treatment -Baseline |  |
| N (Missing) | 104(12) |
| Mean±SD | -7.9±7.0 |
| Mean(95%CI) | (-9.2,-6.5) |
| *t* | 11.52 |
| *P* | <0.0001 |

Comparisons of change from baseline in treatment xx weeks were performed using paired t test.

Supplementary Table 4 The Changes from Baseline in SDS Total Score (FAS)

|  | **Toludesvenlafaxine hydrochloride sustained-release tablets**  **(n=116)** |
| --- | --- |
| Baseline |  |
| N (Missing) | 115(1) |
| Mean±SD | 18.1±6.0 |
| 2-week treatment |  |
| N (Missing) | 115(1) |
| Mean±SD | 14.1±6.8 |
| 2-week treatment -Baseline |  |
| N (Missing) | 115(1) |
| Mean±SD | -4.0±5.9 |
| Mean(95%CI) | (-5.1,-2.9) |
| *t* | 7.34 |
| *P* | <0.0001 |
| 4-week treatment |  |
| N (Missing) | 108(8) |
| Mean±SD | 10.8±6.8 |
| 4-week treatment -Baseline |  |
| N (Missing) | 108(8) |
| Mean±SD | -7.1±6.7 |
| Mean(95%CI) | (-8.4,-5.9) |
| *t* | 11.07 |
| *P* | <0.0001 |
| 8-week treatment |  |
| N (Missing) | 105(11) |
| Mean±SD | 7.9±7.0 |
| 8-week treatment -Baseline |  |
| N (Missing) | 104(12) |
| Mean±SD | -10.1±7.8 |
| Mean(95%CI) | (-11.6,-8.6) |
| *t* | 13.21 |
| *P* | <0.0001 |

Comparisons of change from baseline in treatment xx weeks were performed using paired t test.

Supplementary Table 5 The Changes from Baseline in Q-LES-Q-SF Total Score (FAS)

|  | **Toludesvenlafaxine hydrochloride sustained-release tablets**  **(n=116)** |
| --- | --- |
| Baseline |  |
| N (Missing) | 115(1) |
| Mean±SD | 32.5±7.0 |
| 2-week treatment |  |
| N (Missing) | 115(1) |
| Mean±SD | 39.0±8.7 |
| 2-week treatment -Baseline |  |
| N (Missing) | 115(1) |
| Mean±SD | 6.6±7.2 |
| Mean(95%CI) | (5.3,7.9) |
| *t* | 9.84 |
| *P* | <0.0001 |
| 4-week treatment |  |
| N (Missing) | 108(8) |
| Mean±SD | 42.0±8.7 |
| 4-week treatment -Baseline |  |
| N (Missing) | 108(8) |
| Mean±SD | 9.7±8.2 |
| Mean(95%CI) | (8.1,11.3) |
| *t* | 12.22 |
| *P* | <0.0001 |
| 8-week treatment |  |
| N (Missing) | 105(11) |
| Mean±SD | 46.1±9.6 |
| 8-week treatment -Baseline |  |
| N (Missing) | 104(12) |
| Mean±SD | 13.9±10.0 |
| Mean(95%CI) | (12.0,15.9) |
| *t* | 14.21 |
| *P* | <0.0001 |

Comparisons of change from baseline in treatment xx weeks were performed using paired t test.

Supplementary Table 6 Correlation analysis of neurotrophic factors and symptom improvements (FAS)

|  | **Toludesvenlafaxine hydrochloride sustained-release tablets** | | | |
| --- | --- | --- | --- | --- |
|  | (mBDNF -Baseline)/  Baseline | (pro-BDNF -Baseline)  Baseline | (VEGF -Baseline)/  Baseline | (IGF-1 -Baseline)/  Baseline |
| (8-week treatment -Baseline)/ Baseline |  |  |  |  |
| DARS Total Score | n=99(*r*= 0.06356)[*P*= 0.9861] | n=99(*r*=-0.01035)[*P*=0.9861] | n=99(*r*=-0.07559)[*P*=0.9861] | n=99(*r*=-0.04462)[*P*=0.9861] |
| MADRS Total Score | n=99(*r*= -0.09641)[*P*= 0.9861] | n=99(r=0.10849)[P=0.9861] | n=99(*r*=0.10308)[*P*=0.9861] | n=99(*r*=0.02435)[*P*=0.9861] |
| Anhedonia factor score | n=99(*r*= -0.05845)[*P*=0.9861] | n=99(*r*=0.07153)[*P*=0.9861] | n=99(*r*=0.09560)[*P*=0.9861] | n=99(*r*=0.00177)[*P*=0.9861] |
| SHAPS Total Score | n=99(*r*=0.05809)[*P*=0.9861] | n=99(*r*=-0.03406)[*P*=0.9861] | n=99(*r*=-0.00853)[*P*=0.9861] | n=99(*r*=0.07899)[*P*=0.9861] |
| SDS Total Score | n=99(*r*=0.01447)[*P*=0.9861] | n=99(*r*=-0.04360)[*P*=0.9861] | n=99(*r*=0.03512)[*P*=0.9861] | n=99(*r*=0.10640)[*P*=0.9861] |
| Q-LES-Q-SF Total Score | n=99(*r*=-0.06096)[*P*=0.9861] | n=99(*r*=0.01002)[*P*=0.9861] | n=99(*r*=-0.02439)[*P*=0.9861] | n=99(*r*=-0.23438)[*P*=0.6614] |

Pearson correlation analysis was used to calculate the paired samples, correlation coefficients and FDR Corrected P value.

**Safety**

**Vital signs**

There were no significant changes in armpit temperature or changes in respiration rate or changes in pulse after treatment intra- and inter-group.

**Supine and orthostatic blood pressure**

Toludesvenlafaxine hydrochloride sustained-release tablets might slightly increase the mean (±SD) changes of supine diastolic pressure. The baseline mean diastolic pressure in supine position were 74.3±8.7 mmHg. Post hoc test found that supine diastolic pressure at 8-week time points of follow-up were significantly higher than baseline (P=0.0221). (see Supplementary Table 7).

Supplementary Table 7 The Changes from Baseline in Mean Supine Systolic and Diastolic Pressure

|  | **Toludesvenlafaxine hydrochloride sustained-release tablets (N=123)** |
| --- | --- |
| **Supine Systolic Pressure** |  |
| Baseline |  |
| N (Missing) | 123(0) |
| Mean±SD | 113.0±12.4 |
| 8-week treatment |  |
| N (Missing) | 104(19) |
| Mean±SD | 113.0±12.7 |
| 8-week treatment -Baseline |  |
| N (Missing) | 104(19) |
| Mean±SD | 0.7±11.7 |
| *t* | 0.59 |
| *P* | 0.5598 |
| **Supine Diastolic Pressure** |  |
| Baseline |  |
| N (Missing) | 123(0) |
| Mean±SD | 74.3±8.7 |
| 8-week treatment |  |
| N (Missing) | 104(19) |
| Mean±SD | 76.2±9.8 |
| 8-week treatment -Baseline |  |
| N (Missing) | 104(19) |
| Mean±SD | 2.2±9.7 |
| *t* | 2.32 |
| *P* | 0.0221 |

**Laboratory tests**

Except some individuals were abnormal with clinical significance, laboratory tests didn’t show any significant trend with time changes. Treatment-emergent adverse events of laboratory tests were listed in Supplementary Table 8. All the TEAEs were mild or moderate.

**Supplementary Table 8. Treatment-Emergent Adverse Events of laboratory tests**

|  | **Toludesvenlafaxine hydrochloride sustained-release tablets (N=123)** | | |
| --- | --- | --- | --- |
|  | **Events** | **Cases** | **Incidence (%)** |
| **Blood biochemistry** |  |  |  |
| Aspartate aminotransferase increased | 1 | 1 | 0.8 |
| Aspartate transaminase increased | 1 | 1 | 0.8 |
| hbilirubin increased | 1 | 1 | 0.8 |
| **Urinalysis** |  |  |  |
| Urine albumin detection | 1 | 1 | 0.8 |
| **12-Lead ECG** |  |  |  |
| QT interval was prolonged | 1 | 1 | 0.8 |
| ST segment anomaly | 1 | 1 | 0.8 |
