# Supplementary figures and images for: Efficacy and Safety of Toludesvenlafaxine Hydrochloride Sustained-Release Tablets in Depression With Anhedonia: A Single-Arm, Multicenter Clinical Study
Source: Depress Anxiety. 2025 May 5;2025:6130764. doi: 10.1155/da/6130764 (PMC12069848; doi:10.1155/da/6130764)

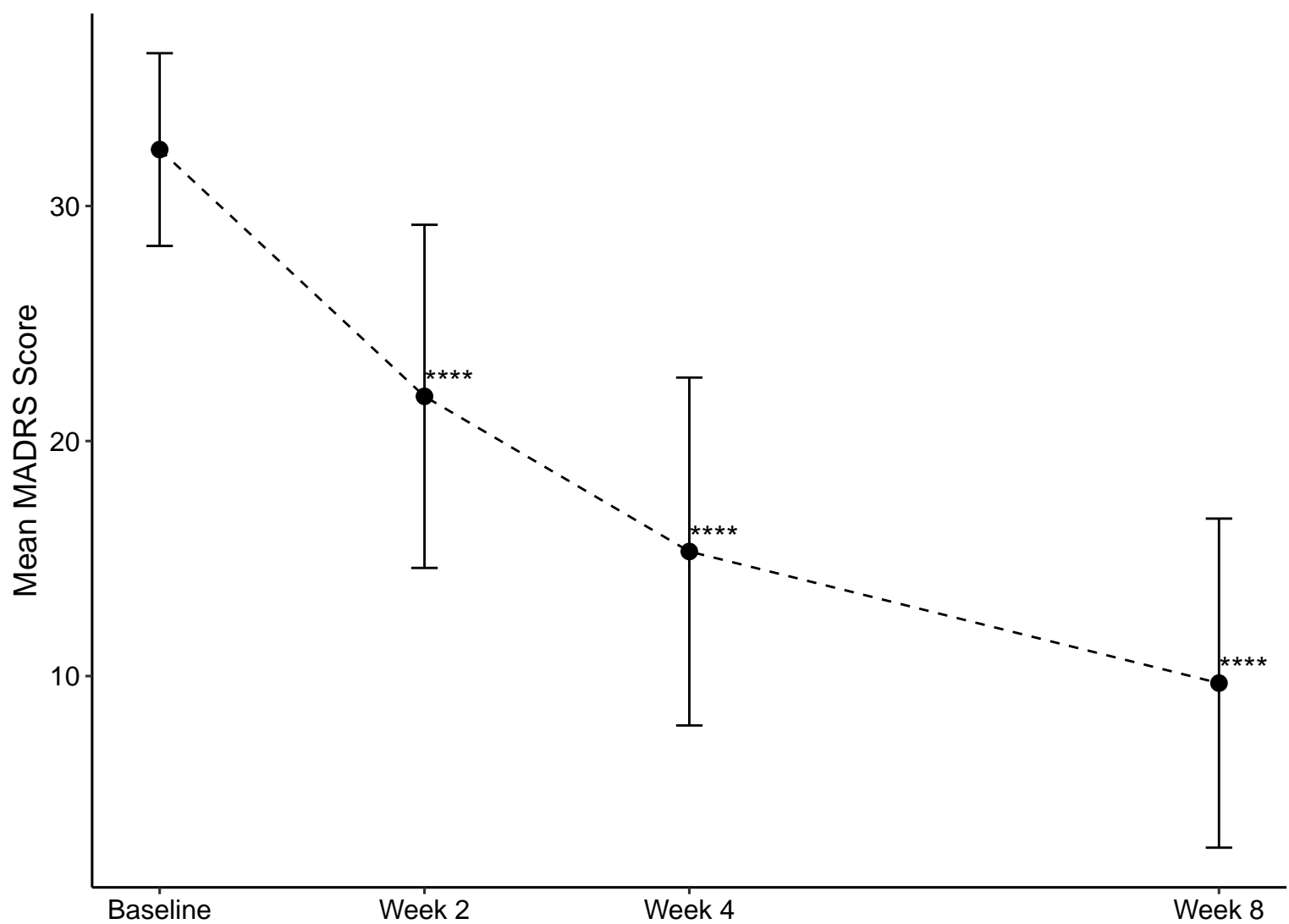

Supplement: Supporting Information 2 — Figure S1. The changes from baseline in MADRS total score (FAS). ⁣∗⁣∗⁣∗⁣∗ indicates p < 0.0001. FAS, full analysis set; MADRS, Montgomery–Asberg Depression Rating Scale. [file 6130764.f2.pdf]

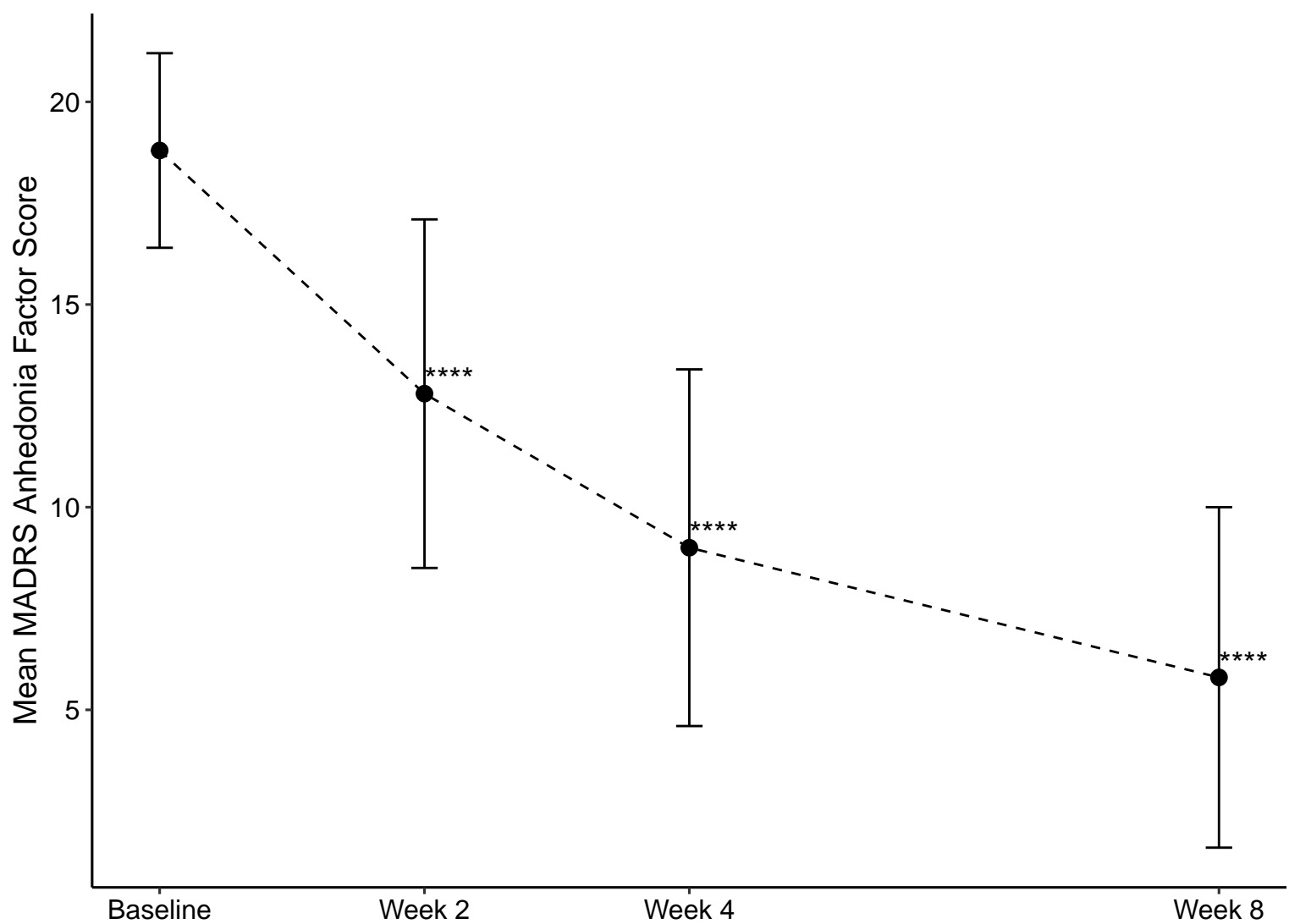

Supplement: Supporting Information 3 — Figure S2. Changes from baseline in MADRS anhedonia factor score (FAS). ⁣∗⁣∗⁣∗⁣∗ indicates p < 0.0001. FAS, full analysis set; MADRS, Montgomery–Asberg Depression Rating Scale. [file 6130764.f3.pdf]

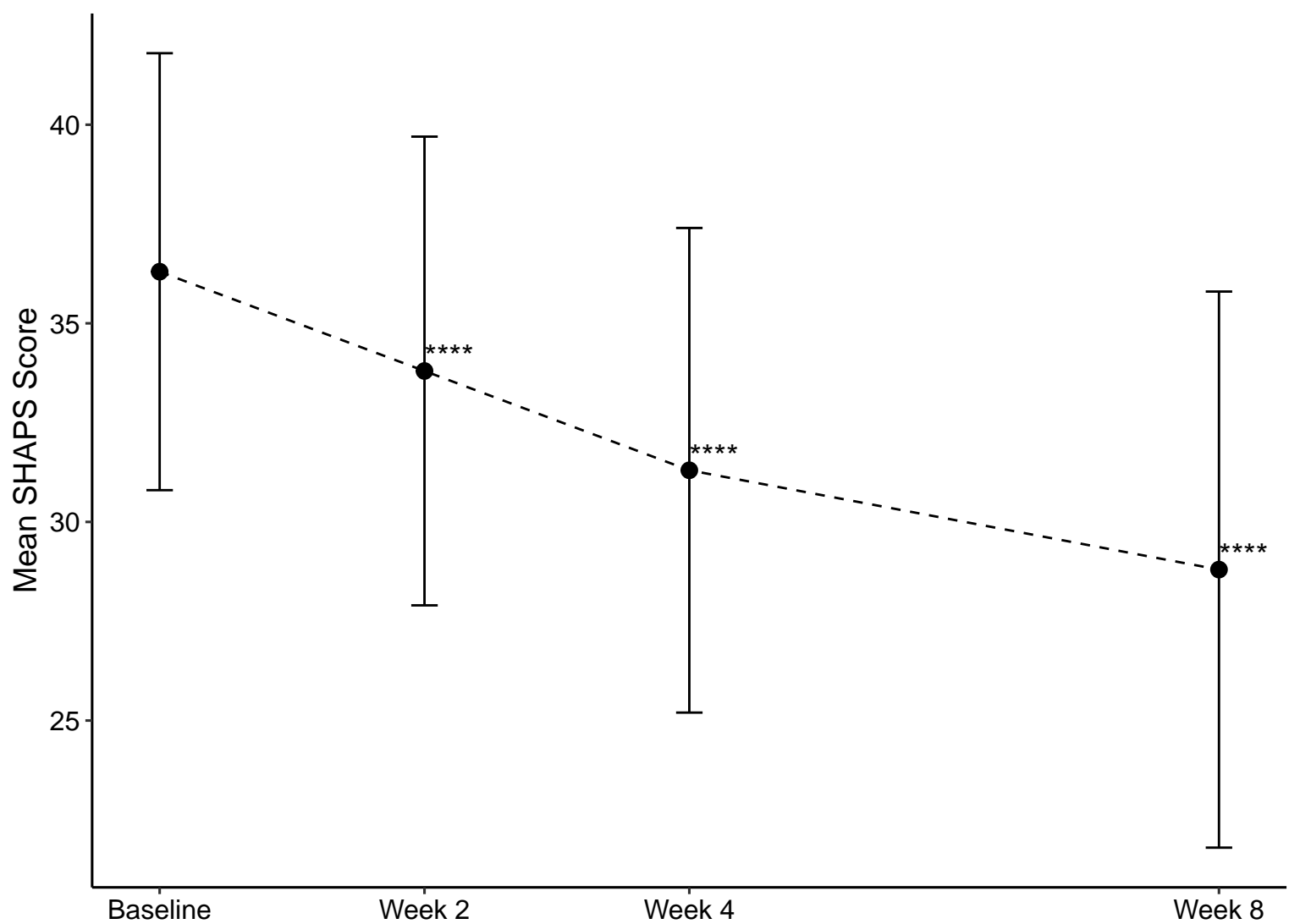

Supplement: Supporting Information 4 — Figure S3. Changes from baseline in SHAPS score (FAS). ⁣∗⁣∗⁣∗⁣∗ indicates p < 0.0001. FAS, full analysis set; SHAPS, Snaith–Hamilton Pleasure Scale. [file 6130764.f4.pdf]
